# Cultivating Compassion and Reducing Stress and Mental Ill-Health in Employees—A Randomized Controlled Study

**DOI:** 10.3389/fpsyg.2021.748140

**Published:** 2022-01-27

**Authors:** Christina Andersson, Christin Mellner, Peter Lilliengren, Stefan Einhorn, Katja Lindert Bergsten, Emma Stenström, Walter Osika

**Affiliations:** ^1^Karolinska Institutet, Stockholm, Sweden; ^2^Center for Arts, Business & Culture (ABC), Stockholm University, Stockholm, Sweden; ^3^Ersta Sköndal Bräcke University College, Stockholm, Sweden; ^4^Department of Psychology, Clinical Psychology, Uppsala University, Uppsala, Sweden; ^5^Stockholm School of Economics, Stockholm, Sweden

**Keywords:** compassion, stress, mental health, organization, intervention

## Abstract

Stress and mental ill-health carry considerable costs for both individuals and organizations. Although interventions targeting compassion and self-compassion have been shown to reduce stress and benefit mental health, related research in organizational settings is limited. We investigated the effects of a 6-week psychological intervention utilizing compassion training on stress, mental health, and self-compassion. Forty-nine employees of two organizations were randomly assigned to either the intervention (*n* = 25) or a physical exercise control condition (*n* = 24). Multilevel growth models showed that stress (*p* = 0.04) and mental ill-health (*p* = 0.02) decreased over 3 months in both groups (pre-intervention to follow-up: Cohen’s *d* = −0.46 and *d* = 0.33, respectively), while self-compassion only increased in the intervention group (*p* = 0.03, between group *d* = 0.53). There were no significant effects on life satisfaction in any of the groups (*p* > 0.53). The findings show promising results regarding the ability of compassion training within organizations to decrease stress and mental ill-health and increase self-compassion.

## Introduction

The global increase in stress and mental ill-health has been put forth as one of the major sustainability challenges for the future, and the global goals for sustainable development include the objective to “promote mental health” (United Nations, 2020 Agenda for sustainable development). The goal of promoting mental health is highly relevant to today’s work organizations; stress and mental ill-health carry considerable costs at both the individual and organizational levels. Apart from individual suffering, mental ill-health costs the global economy $1 trillion in lost productivity per year, according to the World Health Organization (2020). In Sweden the cost of stress (SOU, 2019:5) is high in many organizations, and stress-related mental ill-health is a leading cause of long-term sick-leave (Swedish Social Insurance Agency, 2020).

Both compassion which is directed toward others and self-compassion have shown associations with reduced stress (Pace et al., 2009; Breines et al., 2014); lower levels of mental ill-health (MacBeth and Gumley, 2012; Muris and Petrocchi, 2016; Homan and Sirois, 2017; Matos et al., 2017; Wilson et al., 2019); and increased well-being (Zessin et al., 2015).

Additional randomized controlled intervention studies with longer follow-up periods in organizational contexts are needed, to further evaluate the associations previously found and investigate whether these associations are sustained after compassion training. The present study investigated the effects of a psychological intervention utilizing compassion training on stress, mental ill-health, life satisfaction, and self-compassion among employees of two Swedish organizations.

### Stress, Mental Health, and Work

Stress is not a disease in itself, but is defined as a reaction to threat, and perceptions of threat, which exceeds an individual’s resources (Cohen et al., 1983; Folkman et al., 1986; Ursin and Eriksen, 2010). A stress response includes both biological and psychological components; how it manifests depends upon the nature and duration of the stressor, and upon the individual’s resilience (Lupien et al., 2009). According to the Cognitive Activation Theory of Stress (CATS), the outcome of a stressful situation depends upon an individual’s expectations of being able to cope with that situation, using different coping styles including ranges of hopelessness, helplessness and overcoming (Ursin and Eriksen, 2010). Further research from Brosschot et al. (2018) argues that our mind is always searching for cues of safety, and that a default stress response occurs when an individual perceives their environment as unsafe. Both acute and chronic stress trigger and increase mental ill-health (Grossi et al., 2015) as well as somatic ill-health (Brotman et al., 2007). Both types of stress lead to subsequent reduced working capacity and life satisfaction, respectively (Zuzanek, 1998).

In the context of work, Karasek and Theorell (1990) have outlined a stress model (JDC-S model) of job demands, comprising time pressure and conflicting demands; job control, comprising decision latitude in one’s work; and social support. Their model includes implications for employee stress and mental health. This model has received substantial support; a large body of earlier research has shown that increased risk for developing both fatigue syndrome and depression, as well as the need for long-term sick-leave, have been found among employees who experience high demands and low control in their work (SBU, 2014). Similar increases in mental ill-health have been observed among those who use “hidden coping,” holding back one’s emotions, at work (Norlund, 2011).

### Compassion

Studies have suggested there is a difference between compassion and empathy: the latter represents the capacity to share the feelings of others through knowing that an emotion is not one’s own but another’s, although the emotion resonates with oneself (Singer and Klimecki, 2014; Gilbert et al., 2019a,b). Empathy involves an internal response to another’s happiness or suffering, and awakens similar feelings within oneself, which can lead to distress and eventual empathy fatigue (Singer and Klimecki, 2014; Västfjäll et al., 2014; Håkansson Eklund and Summer Meranius, 2020). Meanwhile, compassion has been characterized by feelings of warmth, concern and care, along with a strong motivation to improve another’s well-being. One definition of compassion is: “the felt response to perceiving suffering that involves an authentic desire to ease distress” (Goetz et al., 2010). A recent review of definitions and measures on compassion (Strauss et al., 2016) has proposed that compassion includes five elements: (1) recognizing suffering; (2) having empathic concern for the person suffering; (3) understanding the universality of human suffering; (4) embracing uncomfortable feelings with non-judgment and emotional warmth; (5) motivation to act to alleviate suffering.

Individuals who report more compassion for others have been found to exhibit more prosocial behaviors, including empathy and forgiveness; and to provide social support to others (Fehr et al., 2009). Compassion directed toward others can act as a buffer against stress; on the receiving end, as experiencing support from others can increase an individual’s capacity to handle difficult challenges and situations that are perceived as unsafe (Schnall et al., 2008, 2010; Cosley et al., 2010; Coan et al., 2013).

### Self-Compassion

Compassion can also be directed toward oneself, which is referred to as self-compassion. This involves a loving, non-judgmental understanding of one’s own shortcomings, placing the perception of one’s suffering and difficulties into a larger perspective of what it means to be human (Neff, 2011). According to Neff (2011), self-compassion includes the following three components: (1) self-kindness rather than self-judgment, i.e., being kind toward oneself when encountering pain and personal shortcomings, instead of ignoring them or hurting oneself with self-criticism; (2) common humanity rather than isolation, i.e., recognizing that suffering and personal failure are parts of the shared human experience; and (3) mindfulness rather than over-identification, i.e., taking a balanced approach to one’s negative emotions so that feelings are neither suppressed nor exaggerated. Self-kindness refers to refraining from criticizing oneself for mistakes or flaws, instead behaving toward oneself with understanding and support. Common humanity involves the recognition that everyone makes mistakes or fails at times, and the acknowledgment that this is part of being human. Being mindful allows an awareness of negative self-talk and identification with one’s feelings and thoughts, which makes it possible to address these emotions with love and compassion. The purpose of self-compassion is to enable “people to suffer less while also helping them thrive” (Neff and Dahm, 2015).

Self-compassion has been found to help generate positive emotions by increasing the capacity to embrace negative ones (Neff et al., 2007; Fredrickson et al., 2008). It has been associated with reducing anxiety, depression, and stress (MacBeth and Gumley, 2012; Ferrari et al., 2019), increasing happiness and self-esteem (Mongrain et al., 2010) and elevating emotional intelligence, wisdom, life satisfaction, and feelings of social connectedness (Neff, 2003).

### Compassion and Self-Compassion in Organizational Settings

Compassion in the organizational setting has been defined as sets of practices among members and employees which build and sustain the health of individuals, teams, and the organization as a whole (Kanov et al., 2004; Lilius et al., 2011a,b). Moreover, Worline and Dutton (2017) describe organizational compassion as a four-part process: (1) noticing that suffering is present in an organization; (2) making meaning from suffering in a way that contributes to a desire to alleviate it; (3) feeling concern for the people suffering; and (4) taking some manner of action to alleviate suffering.

This is in line with the model Atkins and Parker (2012) have developed for individual compassion in organizations, which includes such components as noticing, appraising, feeling, and acting. In their model, psychological flexibility—in which an individual does not try to escape from or control negative experiences, but rather is willing to face them (Fledderus et al., 2010)—enhances these components. Miller (2007) describes establishing compassionate communication in organizations by focusing on processes of noticing, connecting, and responding. Compassion has also been outlined as a framework for building flourishing relationships (Condon et al., 2019), in which compassionate actions have been shown to spread among networks of people (Fowler and Christakis, 2010). Thus, it can be argued that a foundation for compassionate culture is built on psychological safety that arises among groups. This, in turn, can be regarded as crucial for organizations to learn, innovate, and foster individual growth (Edmondson, 1999).

Compassion in the workplace may act as an emotionally uplifting mechanism, because it can transform and regulate difficult emotions and instead motivate employees toward finding joy in feeling connected to others. Indeed, compassion in the workplace alters the “felt connection” between people, and is associated with such positive attitudes and behaviors as justice, responsibility, and kindness (Lilius et al., 2008; Gibb and Rahman, 2018). In addition, individuals’ capacity can increase through embracing adverse events with courage, engagement, wisdom and emotional warmth, instead of with catastrophic thinking, judgment or self-loathing (Tsoukas and Knudsen, 2005). The ability to recognize mistakes without being overwhelmed by negative affect (Leary et al., 2007) has moreover been found to increase individual effectiveness (Reizer, 2019). Developing a capacity for compassion can also function as an antidote to compassion fatigue and empathic distress, particularly in healthcare organizations (Trzeciak and Mazzarelli, 2019). Learning to overcome fear in relation to compassion can increase psychological functioning and help employees become open to compassion from both others and themselves (Kirby et al., 2019). Prior research further proposes that compassion directed toward others can function as a key to organizational performance, for example by establishing pathways to increase learning in organizations (Guinot et al., 2020) or for organizations to be cost-effective (Trzeciak and Mazzarelli, 2019).

Scholars have put forth self-compassion as being of particular interest for organizations (Kirby, 2017), due to it being a coping strategy that transforms such emotions as fear, sadness, anger, guilt, and shame into connection, common humanity, meaning, and hope (McGonigal, 2019). A recent study has shown that organizational leaders improve their emotional regulation skills after undergoing self-compassion training (Paakkanen et al., 2020). Among employees, previous studies have found that self-compassion has positive impacts on psychological strength, job performance (Reizer, 2019), and job satisfaction (Abaci and Ardi, 2013). Prior research has found that self-compassion can act as a resource for coping with uncertain and challenging situations in ways that result in increased professional well-being and reduced work-related exhaustion (Babenko et al., 2019). Self-compassion increases motivation for self-improvement (Breines and Chen, 2012), and improves interpersonal and social relationships (Crocker and Canevello, 2012, 2017). Lastly, self-compassion enhances the quality of team-based relational exchange and compassion toward others by increasing social and psychological safety, in which the latter occurs *via* a person’s sensitivity to prosocial cues and signs of warmth from colleagues (Pinard et al., 2020).

### Compassion-Based Interventions

Both compassion and self-compassion have been outlined as competencies, skills which can be learned (Frost, 1999; Frost et al., 2006; Gilbert, 2019). Related to this connection, there has been a sharp increase during the last 10 years in the number of evaluations of compassion-based interventions. One example among these involves compassion-mind training (Gilbert, 2017), in which a goal is to cultivate compassion in three directions for optimal mental health and well-being: giving compassion to others, receiving compassion from others, and providing self-compassion. Across all these components, compassion can be broadly understood as a cognitive, affective, and behavioral process, which involves acknowledging suffering and developing both intention and motivation to reduce the suffering, by learning to tolerate painful thoughts and feelings, being emotionally touched by them, and feeling social connectedness.

A range of compassion-based interventions have been developed, including Compassion-Focused Therapy (Gilbert, 2014), Mindful Self-Compassion (Neff and Germer, 2013), Compassion Cultivation Training (Jazaieri et al., 2013), Cognitively Based Compassion Training (Pace et al., 2009), and Cultivating Emotional Balance (Kemeny et al., 2012). Many of these focus on the view of compassion as an intrinsic motivation (Gilbert, 2017, 2019), and on the development of affiliative and prosocial functioning (Weng et al., 2013; Kirby, 2017), as well as strengthening mental health (Gilbert and Procter, 2006; Cuppage et al., 2018; Irons and Heriot-Maitland, 2020) and physical health (Austin et al., 2020).

Regarding potential mechanisms, compassion-based interventions use specific strategies aimed at calming and soothing the individual, including practices of breathing, use of friendly voices, and use of facial and bodily expressions. These tactics have been found to decrease stress through activation of the parasympathetic nervous system, which also improves heart rate variability (Krygier et al., 2013). Activation of the parasympathetic nervous system provides feelings of psychological safety, in contrast to activation of the sympathetic nervous system, which occurs when an individual perceives threat or stress (Thayer and Lane, 2000; Liotti and Gilbert, 2011; Klimecki et al., 2014).

In summary, although previous research indicates that compassion and self-compassion are associated with stress and mental health and are further associated with such organizational outcomes as job satisfaction and performance, work engagement, exhaustion, and professional life satisfaction, randomized controlled studies with longer follow-up periods within organizational settings remain limited. The present study aimed to evaluate the effects of a psychological intervention based on compassion training, compared with an active control group treatment program of physical exercise, on employees’ stress, mental health, and life satisfaction. The study evaluated changes in self-compassion in order to determine whether potential intervention effects might be attributed to increases in self-compassion *per se*. The choice of physical exercise as an active control group treatment was based on the evidence that physical exercise can improve stress resilience (Mücke et al., 2018), and that physical exercise is also commonly recommended within organizations as a tactic to decrease employee stress and improve overall health. Specifically, we hypothesized that the compassion intervention would reduce employee stress, anxiety, and depression, and would increase life satisfaction, at similar, or higher, levels to those brought about by physical exercise. We also hypothesized that the compassion intervention would increase self-compassion at higher levels than those brought about by physical exercise.

## Materials and Methods

The study has been approved by the regional ethics committee in Stockholm (Dnr: 2015/1589-31/5) but was not preregistered.

### Procedure and Participants

The principal investigator was independently contacted by the HR departments of the two participating organizations with a request to conduct a stress reduction intervention. Subsequently, the researcher and the relevant leadership of the two organizations made the joint decision to conduct a randomized controlled trial, to enable collection of data to address the research questions.

The respective HR departments at each organization conducted the recruitment of participants, by sending out a call for interest together with written information to enable employees themselves to decide whether they wanted to participate. No compensation was provided in exchange for participation. A key inclusion criterion was that a given participant had felt a subjective experience of perceived stress. No formal assessment of perceived stress was, however, performed due to logistical challenges with collecting such measurements. Informed consent was filled out by all participants. The available sample comprised 49 employees (the participants were between 25–55 years old and 96 percent were women) in two Swedish organizations. One organization is a public agency in the social services field and the other is a private company in the financial sector. Randomization of assigning participants to either the intervention group or control group was conducted utilizing an online tool for research studies: www.random.org. After randomization, two participants allotted to the control group dropped out before filling out the questionnaires at baseline. Hence, 25 participants in the intervention group and 22 participants in the control group filled out the questionnaires at baseline. The final study sample consisted of 14 participants from the private company and 35 participants from the public agency. The intervention was conducted in 2016 and was carried out separately at each workplace.

In the first step, before the intervention commenced, all participants received the same instructions. The participants received this information about the study from the research team delivered by their organization’s respective HR department representatives, who sent this information to the participants’ work email. Further information and instructions regarding the testing were provided to participants on site, where they completed pen-and-paper self-report questionnaires. They did so one at a time, following a schedule: each participant had been given a code to ensure anonymity, and each of the two organizations set aside a specific room at each workplace for the current study where participants completed questionnaires. In the second step, after the baseline assessment, the respective HR departments informed each participant whether they had been assigned to the intervention group or the control group. Both the participants and the researchers were blinded to the group allocation before filling in the baseline questionnaire. The same procedure was conducted for the second and third assessments, which occurred after 6 weeks when the intervention ended, and at 3-month follow-up: participants completed their assessment at a scheduled time in a specific room at each workplace set aside for the present study within both of the participating organizations.

### Intervention

The intervention (developed by first author CA and fifth author KB) consisted of an in-person 6-week structured group format, including weekly 2-h group sessions constituting a secular program to improve stress management, emotional regulation, developing compassion in different directions, gratitude, and wisdom. The program was based on the definitions of compassion developed by Gilbert (2014) and Worline and Dutton (2017). Further, the program was built on the models and exercises within Compassion-Focused Therapy—developing a compassionate self and the three-circle emotional regulation model (Gilbert, 2010)—and within Mindful Self-Compassion (Neff and Germer, 2013). The program incorporated neuroscience; attachment theory; contemplative traditions including mindfulness and loving-kindness; psychodynamic therapy; cognitive behavioral therapy; and affect theory (Kirby et al., 2017). Some exercises, such as mental imagery exercises, were adjusted by the authors CA and KB. The program incorporated all perspectives on stress described above to enable the participants to learn how to (1) transform negative perceptions regarding stress into an empowering view of stress, in terms of their stress-related coping capacity (CATS: Ursin and Eriksen, 2010); (2) search for safety cues in the environment as a coping strategy to regulate stress (Brosschot et al., 2018); and (3) understand the roles of job demands, job control, and social support at work for stress and mental health (JDC-S model: Karasek and Theorell, 1990).

During the program, participants learned various formal and informal practices, including mindful self-compassion meditation and mindful breathing. They also learned reflective and experiential techniques including mental imagery, exercises, and group conversations. Participants were encouraged to practice daily between the weekly program sessions.

The program was delivered by the authors CA and KB, who are clinical psychologists certified within both Compassion-Focused Therapy (Gilbert, 2010) and Mindful Self-Compassion (Neff and Germer, 2013); each possesses more than 5 years of personal practice, and experience in facilitating compassion programs; and have foundations in the academic and practical knowledge regarding the relevant psychological background on which the program was built.

The control group was instructed to perform physical exercise independently during the 6-week compassion intervention period. Hence, the control group participants did not attend a specific training program but were instructed to execute some form of physical exercise by their own choice. Criteria for this activity were that it should increase heart rate, as with powerwalks, and be performed at least three times weekly for 30 min during each of the 6 weeks, or up to 2 h each week. After the intervention, the participants in the control group gave either oral or written information (number of occasions and dates) on their physical exercise during the 6-week intervention period. Evaluation showed adherence to this assignment.

### Outcome Measures

#### Self-Compassion Scale

The present study has used the Swedish translation (Strömberg, unpublished^1^). This 26-item scale (Neff, 2003; Raes et al., 2011) includes three dimensions representing self-compassion: Self-kindness; Common humanity; and Mindfulness, and includes three dimensions representing lack of self-compassion: Self-judgment; Isolation; and Over-identification for which these three subscales are reverse coded. Responses are gathered *via* a five-point Likert-type scale, ranging from *1–Almost never* to *5–Almost always*. Example item: “When I am going through a very hard time, I give myself the care and tenderness I need.” The items were reversed when needing to indicate a high level of self-compassion. Reliability across assessment points in the current sample varied according to Cronbach’s alpha ranging from 0.91 to 0.92.

#### Perceived Stress Scale

The present study has used the Swedish translation (Eskin and Parr, 1996) of the 14-item Perceived Stress Scale (PSS14) (Cohen et al., 1983). Responses are gathered *via* a five-point Likert-type scale, ranging from *0–Never* to *4–Very often*. Example item: “How often during the last month have you felt unable to cope with all the things that you had to do?” The items were reversed when needing to indicate a high level of perceived stress. The total score is 56 and for questions 4, 5, 6, 7, 9, 10, 13 there is a reverse scoring. Reliability across assessment points in the current sample varied according to Cronbach’s alpha ranging from 0.88 to 0.92.

#### Hospital Anxiety and Depression Scale

This 14-item self-report screening scale (Zigmond and Snaith, 1983) rates anxiety and depression in non-clinical populations. It consists of seven items targeting anxiety, and seven items targeting depression. Responses are made on a four-point Likert-type scale ranging from 0 to 3 points, where high scores indicate a high level of anxiety and depression, respectively. Example item: “I feel tense and nervous.” Reliability across assessment points in the current sample varied according to Cronbach’s alpha ranging from 0.86 to 0.93.

#### Satisfaction With Life Scale

This 5-item short scale (Diener et al., 1985) measures global life satisfaction. Responses are made on a seven-point Likert-type scale, ranging from *1–Strongly disagree* to *7–Strongly agree*. Evaluation of the total scores is divided into seven categories: *31–5: Extremely satisfied*; *26–30 Satisfied*; *21–25 Slightly satisfied*; *20 Neutral*; *15–19 Slightly dissatisfied*; *10–14 Dissatisfied*; and *5–9 Extremely dissatisfied*. Reliability across assessment points in the current sample varied according to Cronbach’s ranging from 0.86 to 0.91.

### Statistical Analyses

This study has utilized a randomized design and repeated measurements, taken pre-treatment, post-treatment, and follow-up, within subjects. To account for individual changes over time and test for differences in change rates between the groups, we applied mixed-effects growth curve modeling (e.g., Singer and Willett, 2003).

First, to assess changes in the entire sample across all three measurement points, a basic time-model (Model 1) was estimated for each measure. This model includes random effects for intercept and slope, as well as a fixed effect of Time. Time was coded 0 for pre-treatment, 1 for post-treatment, and 2 for follow-up. Next, a second model (Model 2) was estimated to test for group differences. The group variable, coded 0 for the physical exercise group and 1 for the compassion intervention group, was entered as a fixed effect to control for possible differences in baseline score, as well as in interaction with Time to test for group differences in rate of change across assessment points. To account for possible non-linearity over time, a quadratic term (i.e., *time* × *time*) was tested in both models; however, because no significant quadratic effect was detected, these were discarded for our final models. An unstructured structure was assumed in all models.

Data analyses were conducted according to the intention-to-treat principle: all randomized participants were included, and models were estimated on all available data, using Restricted Maximum Likelihood Estimation (REML). This approach to estimation provides unbiased estimates under the less restrictive assumption that missing data are Missing at Random (MAR; Enders, 2011). Within-group and between-group effect sizes, using Cohen’s *d*, were calculated using model estimates’ observed baseline SDs for the whole sample as recommended by Feingold (2009). In line with Cohen (1992), effects > 0.80 are considered large, 0.50–0.80 moderate, and <0.50 small.

All statistical calculations were performed utilizing the SPSS v.20 software package. Prior to main analysis, all variables were inspected, and met basic assumptions of normality without outliers; thus, no corrections were deemed necessary. The primary significance level was set to 0.05 without correction for family-wise error rate (e.g., Bonferroni), as such correction would arguably be too conservative for the objective of exploring the effects of a novel intervention with a limited sample size (Bender and Lange, 2001).

## Results

Table 1 presents the descriptive results of the study variables for the compassion intervention group and physical exercise group, respectively, at baseline, post-intervention, and 3-month follow-up.

**TABLE 1:** Descriptive statistics.

	Groups			
	Compassion		Active control	
	*n*	M (SD)	*n*	M (SD)
SCS				
Baseline	25	82.7 (17)	22	76.5 (19)
Post	21	89.2 (14)	22	75.8 (19)
Follow-up	16	86.1 (13)	14	72.3 (18)
PSS-14				
Baseline	25	26.8 (11)	22	27.9 (9.1)
Post	21	21.6 (9.8)	22	26.9 (11)
Follow-up	16	23.6 (7.3)	14	26.8 (8.4)
HAD-S				
Baseline	25	11.3 (9.1)	22	12.9 (8.0)
Post	21	8.43 (6.8)	22	11.7 (7.7)
Follow-up	16	9.19 (5.2)	13	11.5 (7.1)
SWLS				
Baseline	25	24.6 (5.9)	22	24.1 (6.8)
Post	21	26.6 (5.9)	21	24.0 (6.9)
Follow-up	16	23.6 (6.2)	13	24.2 (6.2)

*SCS-, Self-Compassion Scale; PSS-14, Perceived stress scale; HAD-S, Hospital Anxiety and Depression Scale; SWLS, Satisfaction with life scale.*

Table 2 displays the results of our multi-level growth modeling. Model 1 indicated that both perceived stress and symptoms of anxiety and depression, significantly decreased over time in the whole sample. Across both groups, the average decrease per assessment point was −2.29 [95% CI: (−4.44, −0.14); *p* = 0.04] for perceived stress (PSS-14), with a pre-follow-up Cohen’s *d* of −0.46. For anxiety and depression symptoms (HAD-S), the estimate was −1.42 [95% CI: (−2.59, −0.25); *p* = 0.02], corresponding to a pre-follow-up *d* of −0.33. When both groups were modeled together, there was no significant change over time in self-compassion (SCS).

**TABLE 2:** Mixed-effects growth models estimating changes over time.

		SCS	PSS-14	HAD-S	SWLS
Model 1	Baseline score				
	Intercept	80.31	27.13	11.93	24.46
	Rate of change				
	Slope	1.36	−2.29*	−1.42*	0.30
	Variance components				
	Residual variance	51.32**	15.50*	7.86**	5.35**
	Intercept	263.49**	95.39**	65.11**	36.21**
	Slope	0.01	34.24	8.56	1.31
Model 2	Baseline score				
	Intercept	76.52	27.91	12.88	23.94
	Group	7.25	−1.48	−1.78	0.98
	Rate of change				
	Slope	-0.75	−1.14	−1.17	0.45
	Time × Group	3.82*	−2.31	−0.52	−0.27
	Variance components				
	Residual variance	47.39**	14.91*	7.46**	5.40**
	Intercept	266.14**	98.21**	66.29**	36.82**
	Slope	0.75	36.89**	9.73*	1.35

**p < 0.01, *p < 0.05.

Model 2 tested for testing between group differences. At baseline, the group estimate was non-significant for all measures, indicating no significant pre-treatment group differences. In terms of rate of change over time, the Time × Group interaction proved significant for self-compassion [SCS; estimate = 3.82; 95% CI: (0.47, 7.17); *p* = 0.03], suggesting that the compassion intervention stimulated growth in self-compassion. Meanwhile, test results suggested the recommended physical exercise did not stimulate growth in self-compassion [estimate = −0.75; 95% CI: (−3.17, 1.68); *p* = 0.53]. The between group Cohen’s *d* at follow-up was 0.51. There were no significant group differences in perceived stress [PSS-14; estimate = −2.31; 95% CI: (−6.65, 2.03); *p* = 0.28] or anxiety and depression symptoms [HAD-S; estimate = −0.52; 95% CI: (−2.93, 1.91); *p* = 0.66]. Nevertheless, estimates favor the compassion intervention with small between group effect sizes (*d* = 0.23 and *d* = 0.12). All estimates of change in life satisfaction (SWLS) were non-significant in both models.

## Discussion

In this randomized controlled study, we evaluated effects of a psychological intervention based on a compassion program, compared to an active control condition involving physical exercise, on employee stress, mental ill-health, self-compassion and life satisfaction. The findings showed a significant, small to moderate decrease in perceived stress, and in symptoms of anxiety and depression, in both groups at follow up, but showed no significant increase in life satisfaction in any of the groups. Importantly, the results showed that only the compassion intervention group demonstrated a significant and moderate increase in self-compassion.

These results align with previous studies implying self-compassion is a coping strategy that can be developed through training (Neff and Germer, 2013; Kirby et al., 2017; Ferrari et al., 2019; Craig et al., 2020; Kotera and Van Gordon, 2021). Thus, when an individual possesses the knowledge and training regarding how to quickly reduce a physiologically stressful response to an emotionally challenging experience, through establishing compassion and confidence within themselves, acting on that knowledge builds resilience to the condition called “empathy distress,” which has been shown to lead to reduced motivation and engagement as well as increased sick-leave (Figley, 1995). Developing self-compassion can be said to counter the subsequent risks from being overly involved, through including oneself in the concern, care, and motivation to improve another’s well-being, or compassion (Singer and Klimecki, 2014). This line of reasoning is further supported by a recent study showing that developing compassion can act as an antidote to compassion fatigue and empathic distress (Trzeciak and Mazzarelli, 2019). This increased capacity for self-compassion could, in turn, be argued to decrease the risk for mental ill-health, as previous findings have shown a negative association between self-compassion and mental ill-health (MacBeth and Gumley, 2012).

Considering the negative effects of mental health problems in the workplace, both regarding individual suffering and organizational costs, effective interventions are indeed needed. Because previous studies on compassion interventions have mainly involved healthcare professionals or employees working in caring-focused environments, evaluating the impacts of compassion training in other work contexts is important (Kotera and Van Gordon, 2021). The findings of the present intervention study contribute to existing knowledge by focusing on professionals in both a public service agency and a private company in the financial sector.

### Strengths, Limitations, and Suggestions for Future Research

Strengths of this study include that the intervention took place at the workplace level; it was a randomized controlled trial; the control group was active; and both participants and researchers were blinded to group allocation. The compassion intervention was also facilitated by trained psychologist and therapists, which is a requirement for conducting this particular intervention. Our findings indicate that the compassion intervention was not significantly more effective than physical exercise on perceived stress, mental ill-health, or life satisfaction. Nevertheless, the small effect sizes in favor of the compassion intervention regarding stress and mental ill-health suggest this may be due to the limited sample size. Since the sample was non-clinical, effect sizes might be expected to be small, which should be taken into consideration for the planning of future studies. Another potential interpretation is that the compassion intervention did not target these outcomes as operationalized and measured by the scales used in the present study. Regarding perceived stress, the compassion training might target other types of stressors, such as emotional suffering. For mental ill-health, it might be the case that the scale used did not capture more subtle changes in mental health; it was developed for clinical populations, while the present study included healthy employees in the working population. Regarding the non-significant finding for life satisfaction in both groups, this might be related to the intervention having been designed to target work-related situations, whereas the scale used mainly reflects individuals’ personal life experiences and perceptions. The follow-up period of 3 months might be too short to capture changes in life satisfaction; one might speculate that a longer follow-up period could have resulted in a larger effect. For instance, Ferrari et al. (2019) have found that self-compassion has a small effect size, but a nevertheless significant effect, on life satisfaction.

This study also includes some limitations. Recruitment of participants was conducted by the respective HR departments of the participating organizations and was not supervised by the research team, therefore no screening was done before the start of the intervention besides the inclusion criteria that participants were to have felt a subjective experience of perceived stress. The small sample size and corresponding low statistical power may reduce the accuracy, reliability, and generalizability of the results. Because this was a first, exploratory test of the novel compassion intervention we did not apply correction for family-wise error rate; thus, results should be interpreted with caution. There was only limited information collected regarding sociodemographic factors, aside from gender. Longer follow-up periods, for example 6 and 12 months, would have been valuable to investigate potential long-term impacts of the compassion intervention as well as larger samples to effectively test if the compassion intervention outperforms physical exercise as an active control. Longer periods would also be beneficial when measuring adherence and engagement in both the intervention and control groups. Lastly, four participants dropped out between baseline and post-intervention in the compassion intervention group. In the control group, there were no dropouts. However, no significant differences were observed between the dropouts and the other participants regarding either baseline levels, gender or organizational affiliation. Because the models were estimated using Restricted Maximum Likelihood Estimation (REML), which provides unbiased estimates under the assumption of MAR (Enders, 2011), the dropouts and missing data are unlikely to have significantly impacted the results.

In future intervention studies investigating compassion within organizations, it would be of interest to include additional groups; for example, dividing a larger sample into four groups, where two would undergo either compassion training or physical exercise, one would undergo both compassion training and physical exercise, and the fourth would be a non-active control group placed on a waiting list for the intervention proven to be most effective. This approach would enable investigation of potential interaction effects and further effects of compassion training—for example, whether such training only results in increased physical exercise, as shown by Horan and Taylor (2018). Future studies would also benefit from including all employee levels within the organization, such as managers. Studies would also benefit from incorporating such key organizational outcomes as turnover, sick-leave, innovation, performance, collaboration, engagement, and quality of service from the perspectives of both the company and its customers or clients. Outcome measures could be targeted toward working conditions, such as job demands, job control, social support, job satisfaction, and work-related stress. Finally, compassion within organizations could be studied simultaneously at both the individual and collective levels; first, (self-)compassion can be regarded in the individual as both capacity and responsibility, and second, it could be argued that these individual-level factors need to be lifted to the collective and organizational levels in order to address potential structural problems in an organization where compassion is lacking.

### Conclusion

The results of this study have shown that both the compassion intervention and physical exercise reduced employees’ stress and mental ill-health. However, only the compassion intervention increased employees’ self-compassion. This can be considered as crucial to both employees and organizations, as previous findings have shown associations between self-compassion and multiple work-related outcomes: for example, psychological strength and job performance (Reizer, 2019); job satisfaction (Abaci and Ardi, 2013); coping successfully with uncertain and challenging situations; increased professional well-being; and reduced work-related exhaustion (Babenko et al., 2019). Further related findings have included enhanced resilience (Delaney, 2018); increased self-improvement motivation (Breines and Chen, 2012); improved interpersonal and social relationships (Crocker and Canevello, 2012, 2017); and improved quality of team-based relational exchange and compassion toward others, through increases in trust and the feeling of social safety (Pinard et al., 2020), and through increased healthy self-care practices (Horan and Taylor, 2018). The findings of the present study can be considered an important contribution to the notion that psychotherapeutic-based interventions in the workplace can improve employee well-being (Goetzel et al., 2018).

## Data Availability Statement

The raw data supporting the conclusion of this article will be made available by the authors, without undue reservation.

## Ethics Statement

The studies involving human participants were reviewed and approved by the regional ethics committee in Stockholm. Dnr: 2015/1589-31/5. The patients/participants provided their written informed consent to participate in this study. Written informed consent was obtained from the individual(s) for the publication of any potentially identifiable images or data included in this article.

## Author Contributions

CA, WO, and SE designed the study and acquired funding. CA sampled the data. PL analyzed the data in collaboration with CA. CA, WO, SE, PL, ES, CM, and KLB contributed to the article and approved the submitted version. All authors contributed to the article and approved the submitted version.

## Conflict of Interest

The authors declare that the research was conducted in the absence of any commercial or financial relationships that could be construed as a potential conflict of interest.

## Publisher’s Note

All claims expressed in this article are solely those of the authors and do not necessarily represent those of their affiliated organizations, or those of the publisher, the editors and the reviewers. Any product that may be evaluated in this article, or claim that may be made by its manufacturer, is not guaranteed or endorsed by the publisher.

## Footnotes

1 Strömberg, B. (2012). *Not Published Thesis. Psychology Institution*. Lund: Lund University.
